# Cat owners’ perception on having a pet cat during the COVID-19 pandemic

**DOI:** 10.1371/journal.pone.0257671

**Published:** 2021-10-20

**Authors:** Tadeusz Jezierski, Irene Camerlink, Rachel S. E. Peden, Jen-Yun Chou, Patryk Sztandarski, Joanna Marchewka

**Affiliations:** 1 Department of Animal Behaviour and Welfare, Institute of Genetics and Animal Biotechnology of Polish Academy of Sciences, Jastrzębiec, Poland; 2 School of Natural and Environmental Science, Newcastle University, Newcastle, United Kingdom; 3 Swine Teaching and Research Center, School of Veterinary Medicine, University of Pennsylvania, Philadelphia, Pennsylvania, United States of America; Universidade do Porto Instituto de Biologia Molecular e Celular, PORTUGAL

## Abstract

Restrictions during the COVID-19 pandemic may affect the lives of pet animals. We aimed to assess the impact of the COVID-19 pandemic on the welfare of pet cats across countries, as well as the owners’ experience in relation to their cat(s). An online survey containing 21 questions was distributed internationally. Questions were related to information about the cat and its behaviour and health, changes in the household due to the pandemic, and how the owner related to the cat. The survey was completed by 324 respondents from 25 different countries. The survey showed that keeping pet cats during the COVID-19 pandemic did not create problems for the owners, except some difficulties in acquiring veterinary care. The majority (67.3%) of respondents reported no changes in their cats’ behaviour. When behavioural changes were reported, they were mostly of a positive nature. Owners who took more measures in relation to the cat to prevent the spread of the virus, e.g., reduced contact, were 1.8 times more likely to report changes in the behaviour of their cats but also 3.8 times more likely to report difficulties related to the care of the cat during the pandemic. Two-third of the respondents indicated a reduction of their own psychological tensions due to having a cat during the pandemic. We concluded that the welfare of pet cats, as reported by the owners, was not adversely affected by the pandemic and the human-cat interaction had positive effects on the owners during the lockdown.

## Introduction

During the COVID-19 pandemic, control measures like quarantine, lockdown and social distancing, may affect not only humans but also their companion animals [1, 2]. One concern is that pets, in particularly cats [3], can be infected by humans and become transmission vectors of SARS-Cov-2 both to conspecifics and to humans [4]. Research published shortly after the outbreak of the COVID-19 pandemic suggested that the likelihood of COVID-19 transmission from pets to humans was minimal [1, 5, 6], but the media may have prompted a societal reaction that resulted in many pet animals being euthanised and thousands being abandoned [2, 7]. Recently, information is accumulating that cats can be susceptible to infection with COVID-19, and that the SARS-Cov-2 virus replicates efficiently in cats and ferrets [3, 4, 8]. Some governments (e.g., the Italian Health Ministry) recommended that people infected with COVID-19 should restrict their contact with pets, until more information is known about the virus [5]. This may result in a change of humans’ attitudes towards pet cats, for example, by keeping physical distance from cats. Therefore, there is a need for more insight in how the pandemic has impacted cat welfare, especially from a global perspective.

Human-animal interactions are an important source of social support for humans (reviewed by [9]). Bowen et al. [1] found that the quality of life of pet owners was strongly influenced by the lifestyle changes and emotional effects of the confinement under quarantine, and that pets provided them with substantial support to mitigate those effects. Oliva and Johnston [10] found that cat ownership was not a significant predictor of loneliness scores during lockdown, however, qualitative insights suggested that cat owners perceived their experience of the lockdown as easier by having a pet.

At the time that this study commenced, no other studies were published yet on the potential changes in cats’ welfare. The aim of this study was therefore to assess the impact of the COVID-19 pandemic on the welfare of pet cats, as reported by the owners, as well as the experience of the owners in relation to their cat, through an international online survey. We hypothesize that during the pandemic cats will show behavioural changes that are perceived by their owners, either positive or negative, and that the owners will perceive their cats(s) as a source of support.

## Materials and methods

### Survey

An anonymous, voluntary and approximately 10-min survey was designed to include questions on potential changes in the welfare of cats during the COVID-19 pandemic. The survey was similar in its structure and focus points to an earlier published survey on dog welfare [11]. The survey did not collect any personal identifier data (i.e., name, email or IP address) and therefore did not require ethical approval from the human or animal ethical review committee in the country of the main authors (Poland). As guidelines for human ethics regarding surveys varies between countries, we performed the survey in accordance with the basic internationally prevailing guidelines/regulations. The survey started with a brief introduction that explained the aim of the project, information on anonymity and data protection of participation as well as a request to confirm the consent to the conditions of participation. To start the survey, participants had to confirm that they had read the aim and conditions of the survey and that they are at least eighteen years old. Informed consent was obtained from all participants at the start of the survey. Twenty-one questions were asked, divided over four parts of the questionnaire: 1) Residence place and information about the cat(s); 2) Changes in the cat’s care, behaviour and health during pandemic; 3) Difficulties and/or advantages of having a cat during pandemic; and 4) The owner and household. The most important questions are provided in the results section along with the outcomes. The full list of questions is provided in S1 File. The survey was available in English, Mandarin (Simplified Chinese), Italian, French, Spanish, German, Portuguese, Dutch and Polish. The survey was placed online using the EUSurvey platform, version v1.5; a free online survey-management system supported by the European Commission. The survey was aimed at cat owners in any country. The survey was advertised by email to research contacts and announcements on social media (on websites and newsletters of animal science societies and networks, Twitter, and on the YouTube channel Animal Welfare Science). The survey was available online from 1 May to 30 June 2020.

### Data preparation and statistical analyses

The data are available online at http://dx.doi.org/10.17632/49v62g8whd.1

Survey responses were first checked for completeness, correctness (i.e., whether respondent birth year was realistic) and whether the respondents were at least 18 years of age. Surveys were 100% completed by 93.5% of the respondents. The remaining respondents did not answer the questions on either the cat’s breed, arrival of the cat and/or on the respondent’s age. Two respondents did not disclose their gender. No surveys were discarded due to incompleteness.

As the number of respondents per country was unequally distributed and relatively low, country was included in the models as a random variable (instead of a predictor variable) to account for similarities between respondents from the same country, e.g., in culture, pandemic restrictions, etc.

Data were analysed using SAS software, version 9.4 (SAS Institute Inc., Cary, NC, USA). The experimental unit was the respondent (i.e., household). Respondents were categorised by ‘restriction’, indicating whether they had been in lockdown, in quarantine or none of these (control). Data are presented as percentages of the number of respondents, and as means with standard deviation (SD) unless stated otherwise. Chi-square tests (Goodness of Fit, one sample test of proportions) were used to examine associations between categorical variables.

The variables ‘Cat behaviour change’ (yes/no), ‘Owner Disadvantages’ (yes/no) and ‘Owner Advantages’ (yes/no) were analysed in generalized linear mixed models (GLIMMIX procedure) with a binary distribution and logit link. The initial predictor variables were: restriction (lockdown / quarantine / control), whether there were children in the household (yes/no), the age category of the cat(s) (kitten / adult / old cat / and the possible combinations between them), arrival in household (<1 month / 1–6 months / 7–12 months / > 1 year before the pandemic), whether restrictive measures (e.g. restricted contact or restricted access to areas) were taken regarding cat(s) to prevent the spread of COVID-19 (yes/no), and whether it was a single- or multiple cat household (yes/no). Country was included as the random variable. The model was stepwise reduced by removing variables with *p*>0.10, until the best model fit was achieved as assessed through the Akaike Information Criterion (AIC) (obtained using ‘method = laplace’). For the details on the full versus reduced models see S2 File. The data are presented as odds ratios (OR) with their confidence intervals (CI).

## Results

### Respondents’ demographics

**Fig 1:**
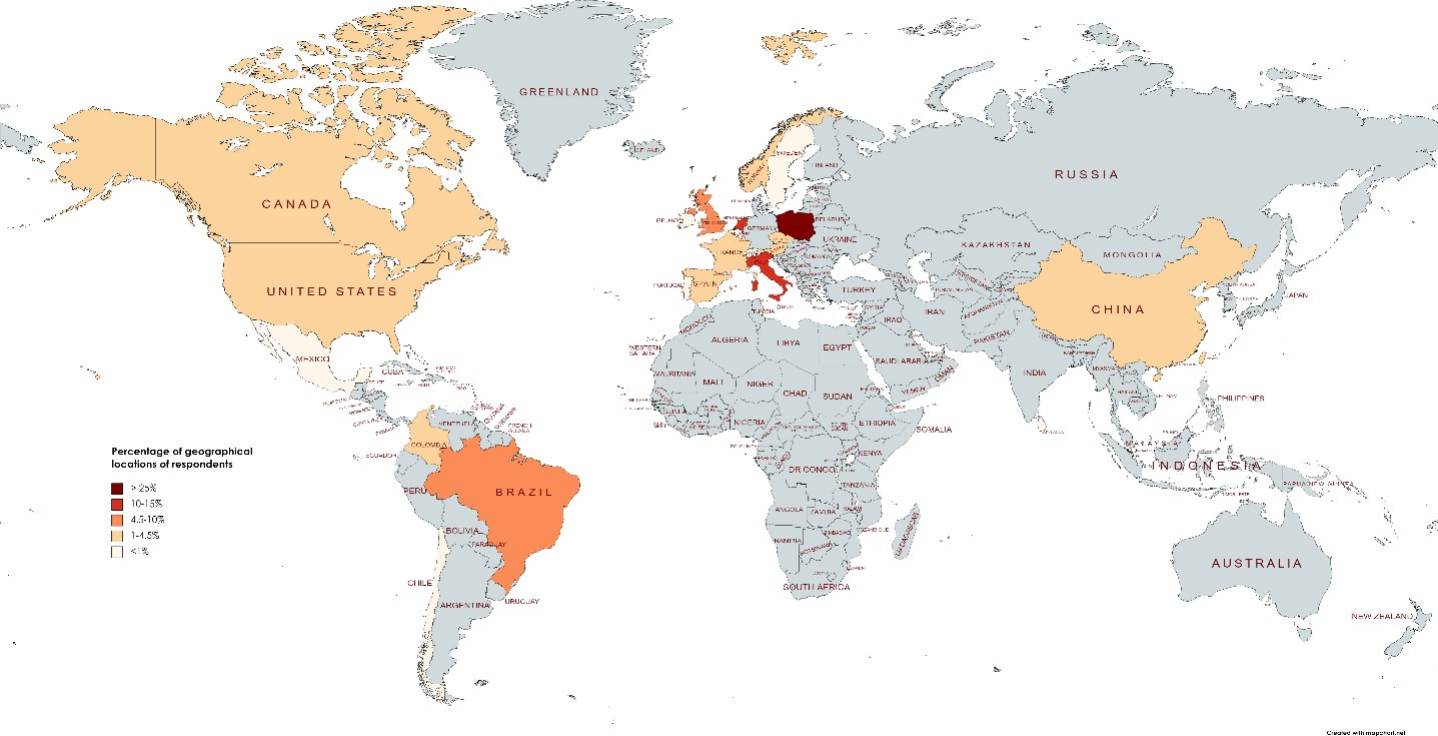
Distribution of respondents across countries.

The survey was completed by 324 respondents. Responses originated from 25 different countries, of which five accounted for 74% of the responses. These were Poland (33%, n = 107), the Netherlands (17%, n = 56), Italy (12%, n = 38), United Kingdom (7%, n = 22) and Brazil (5%, n = 16). Other regions with more than five respondents were France (3.7%, n = 12), China (2.8%, n = 9), Taiwan (2.8%, n = 9), Austria (2.5%, n = 8), Czech Republic (2.5%, n = 8) and Spain (1.9%, n = 6), see Fig 1. The demographics of the respondents are provided in Table 1. The average age of the respondents was 39 ± 12 years (19 respondents did not complete this last question). Up to June 2020, when the survey was closed, 63% of the respondents had been in lockdown for some time, mostly two months (Table 1). Only 10% of the respondents had to go in quarantine, of which 5% went in quarantine for 2 weeks and the other 5% for more than 2 weeks.

**Table 1:** Percentage and number of respondents in each demographic category.

Respondent demographics	% of respondents (n)
Gender	
Male	11.7 (38)
Female	87.6 (284)
Locality	
Countryside	18.7 (53)
Town or suburbs	26.5 (75)
City	21.9 (62)
Capital city	32.9 (93)
Accommodation type	
Apartment or house with a small garden	42.0 (76)
House with large garden or farm	30.9 (56)
Flat or apartment without garden	27.1 (49)
Household composition	
1-person	19.2 (61)
2-person	50.5 (160)
3-person	16.1 (51)
4-person	7.9 (25)
>4-person	5.7 (18)
Children under care	
No	79.9 (259)
Infants of < 1 years old	1.9 (6)
children of 1–5 years old	3.4 (11)
children of 5–15 years old	13.0 (42)
Combination of children from 1–15 years old	1.8 (6)
Respondent in lockdown	
No	27.5 (89)
2 weeks	5.6 (18)
1 month	12.7 (41)
2 months	36.1 (117)
3 months	10.8 (35)
>3 months	7.4 (24)
Respondent in quarantine	
No	89.5 (290)
2 weeks	5.3 (17)
>2 weeks	4.9 (16)

The total number of respondents per question varies as questions were not compulsory and the question on locality and housing type was one multiple choice question which for the analyses was split in two.

### The cats

Respondents mostly had one adult cat of mixed breed, which they had for at least a year before the start of the pandemic (Table 2). Cats were classified as mixed breed cats (53.1%, n = 161, including those where the owner indicated that the cat had no specific breed) and shorthair cats (28.1%, n = 85; of which by 59 respondents referred to as European shorthair). From the 53 owners with purebred cats, 17 had a Maine Coon and six a Siamese cat. Overall, cats mostly stayed indoor during the pandemic.

**Table 2:** Responses related to questions regarding the cats.

**Responses related to the cats**	**% of respondents (n)**
Cats per household	
One	48.2 (156)
Two	30.9 (100)
Three	7.1 (23)
More than three (max. 10)	13.9 (45)
Cat breed	
Shorthair	28.1 (85)
Longhair (breed unspecified)	1.3 (4)
Registered breed	17.5 (53)
Mixed breed	53.1 (161)
Cat’s age (of total number of cats, n = 676)	
Kitten (< 6 months)	12.3 (83)
Adult	74.6 (504)
Old (>12 years)	13.2 (89)
Arrival of the most recent cat to the household	
< 1 month before the pandemic	4.1 (13)
1–6 months before the pandemic	6.6 (21)
7–12 months before the pandemic	11.3 (36)
>1 year before the pandemic	78.1 (250)
Place of the cat(s) during the pandemic	
Outdoor	9.4 (30)
Indoor, but not on places usually occupied by humans	4.9 (16)
Indoor, including places usually occupied by humans	85.8 (278)
Frequency of physical contact with the cat(s) during the pandemic	
Only minimal touching, stroking or petting	4.3 (14)
1–2 times a day briefly touching, stroking or petting	4.6 (15)
Multiple times a day touching, stroking or petting	91.1 (295)

The total number of responses per question varies as questions were not compulsory.

### Changes in cat care in response to the COVID-19 pandemic

Respondents were asked whether they took measures related to their cat(s) in relation to the pandemic. One or more measures were taken by 21% of the respondents, with 79% of the 324 respondents taking no measures related to the cat’s care (Table 3). For two respondents, the cat had to leave the household; one cat was moved to a shelter and another cat was taken care of by acquaintances. There was no correlation between the number of weeks cat owner(s) were in the lockdown and the number of measures (summed) taken in relation to the cats (r_p_ = -0.02; P = 0.66).

**Table 3:** Measures taken in relation to the cat to prevent the spread of COVID-19.

Did you take any special measures or actions with respect to your cat(s) in order to prevent spreading of COVID-19?	% of 324 respondents (n)^1^
No	79.0 (256)
Avoiding the cat(s) to contact other people	13.3 (43)
No more outdoor access beyond the boundaries of the house / garden	7.1 (23)
Avoiding of close contact with other animals	5.6 (18)
Letting the cat(s) less frequently outdoors	2.8 (9)
Disinfection of paws/coat	1.9 (6)
Other	1.9 (6)
Decreasing physical contact with the cat(s) compared to before	1.5 (5)
Leaving of your cat(s) temporarily under custody of other people	0.3 (1)
Giving up the cat(s) forever	0.3 (1)

Percentage of the 324 respondents who answered the question, indicating whether they took the following measures (multiple choice pre-set answers) regarding their cat(s) to prevent the spread of COVID-19.

^1^ As respondents could select multiple choices, the percentage exceeds100%.

### Effects of the pandemic on cat behaviour and health

A third of the respondents (33%) noticed changes in the behaviour of their cat during the pandemic. These changes were mostly positive, such as being calmer and more playful (Table 4). A behavioural change in the cat was more likely to be reported when the owner took restrictive measures (Table 5; *p* < 0.05). There was no significant influence of restrictions and whether the cat arrived less than a month before the start of the pandemic, but it improved to model fit (Table 5). Other variables also did not relate to the reporting of a behavioural change. For 9% (n = 30) of the respondents their cat’s health or appetite changed during the pandemic. Reported changes were increased appetite (n = 10), ‘other’ health issues (n = 10), decreased appetite (n = 4), diarrhoea (n = 3), skin problems (n = 2), constipation (n = 1) and decreased mobility (n = 1). Nineteen percent of 320 respondents had a cat with pre-existing chronic conditions: 26 cats had internal illness, 4 had a skin condition or parasites, and 30 cats had other chronic illnesses.

**Table 4:** Cat’s behavioural changes as reported by the owners.

Did you observe any changes in the behaviour of your cat(s) during the pandemic as compared to before the pandemic?	% of 324 respondents (n)^1^
No	67.3 (218)
More frequent seeking close contacts (approaching, etc.)	21.6 (70)
More playful	12.3 (40)
More frequent vocalizations	10.5 (34)
Calmer	7.7. (25)
More frequent requests for going outdoors	5.2 (17)
Restlessness	2.5 (8)
Anxious	2.2 (7)
Less frequent vocalizations	1.5 (5)
Other	1.5 (5)
Increased damaging behaviour (e.g. scratching)	1.2 (4)
Incontinence / urinating or defecating indoors	0.9 (3)
Apathetic (unresponsive)	0.9 (3)
Reluctance of being let outdoors	0.6 (2)
Avoiding a close contact to caretaker (frequent withdrawal, hiding)	0.6 (2)
Development or increase of repetitive (stereotypic) behaviour	0.6 (2)
Less playful	0.3 (1)
Increased aggression towards humans	0

Percentage and number of the 324 respondents that indicated behavioural changes in their cat(s) (multiple choice pre-set answers).

^1^ As respondents could select multiple choices, the percentage exceeds100%.

**Table 5:** Odds ratios (OR) and their confidence intervals (CI) for the differences between categories within the three main variables.

Variable	Reference class	OR	CI	*p*-value
**Behavioural change in cat**				
Restrictions				0.10
Measures taken	No measures taken	1.825	1.009–3.304	0.046
Arrival of cat in household				0.35
**Difficulties of having a cat**				
Measures taken	No measures taken	3.848	2.066 7.169	<0.001
Arrival of cat in household				0.06
*Arrival <1 month ago*	*Arrival >1 year ago*	1.175	0.309–4.466	
No children in household	Children in household	0.637	0.313–1.298	0.21
**Advantages of having a cat**				
Children in household	No children in household	0.511	0.250–1.046	0.07

Only outcomes of variables that were included in the final model are given, whereas only those of *p*<0.05 and with a (lower boundary) OR >1 were considered significant.

### Difficulties and advantages of having a cat during the pandemic

About a quarter of the respondents (26.9%, n = 87) indicated experiencing difficulties in the care of their cat during the pandemic. The most common difficulty, experienced by 20% of the respondents, was the access to veterinary care (Table 6). The experience of difficulties in cat care was related to whether restrictive measures were taken to avoid the spread of coronavirus through the cat (*p*<0.001; Table 5), with those who took measures related to their cat to prevent the spread of the virus having a 3.8 times higher likelihood of experiencing difficulties in caring for the cat.

**Table 6:** Difficulties in cat care and advantages of having a cat during the pandemic.

Which difficulties did you experience of keeping a cat during the pandemic?	% of 324 respondents (n)^1^
No difficulties	73.1 (237)
More difficult access to veterinary care	19.8 (64)
Food supply	8.0 (26)
Fear of being infected with COVID-19 because of any reason related to the cat	2.5 (8)
Unfavourable changes in the cat’s behaviour	0.9 (3)
Other difficulties	1.2 (4)
**Did you see any advantages of the presence of a cat during the pandemic?**	**% (n)** ^**1**^
Yes, reduction of own psychological tensions because of contact with the cat(s)	75.3 (244)
Yes, desirable changes in the cat’s behaviour	10.8 (35)
Other advantages	11.1 (36)
No advantages	15.4 (50)

Respondents’ answers (multiple choice pre-set choices) to difficulties they experienced due to keeping a cat during the pandemic.

^1^ As respondents could select multiple choices, the percentage exceeds100%.

The majority of the respondents, 84.6% (n = 274) indicated experiencing benefits of having a cat during the pandemic. The most mentioned advantage was a reduction in their own mental tension due to the presence of the cat (Table 6). None of the predictor variables significantly influenced whether respondents indicated advantages, including the level of restrictions (*p* = 0.21) or whether there were children in the household (Table 5).

## Discussion

The aim of the current study was to assess the impact of the COVID-19 pandemic on the welfare of pet cats as reported by the owners, as well as the experience of the owners in relation to their cat(s), by way of an international online survey. The results suggested that the presence of cats served as a mental support to their owners and that the behaviour and health of the cats, as reported by the owners, was overall not negatively affected.

We found that most cat owners did not observe any changes in the behaviour of their cats during the pandemic. For one-third of respondents who observed changes in behaviour, the changes were usually positive, such as being more calm and playful. Consistent with a similar survey in Spain by Bowen et al. [1], most respondents reported no change or a positive change, including more attention-seeking by the cat (36.4% of the respondents in [1]). The positive behaviours of cats could have been influenced by the greater presence of the owners in the house. Research shows that domesticated cats establish close relationships with their owners and expect commitment and attention from them [12]. However, the behaviour of individual cats may be due to their peculiar personality and upbringing [13, 14]. Concerning the amount of handling, this also affects a cat’s attachment to humans [15]. Under experimental conditions, cats which had been handled for 40 minutes a day stayed significantly longer with the test person in a holding test than cats handled for only 15 minutes a day [16]. It is also possible that due to the longer time the owners spent at home during the lockdown, there was a higher probability for them to observe their cats for any changes in their behaviours.

The level of restrictions during this initial COVID-19 wave (May-June 2020), i.e., lockdown, quarantine or no restriction, did not influence the responses of the respondents on the questions related to behavioural changes in the cat, difficulties in cat care or advantages of having a cat. The level of restriction did improve the model fit for behavioural changes, despite not having a significant influence (*p* = 0.10). While 63% of the respondents were or had been in lockdown, only 10% had been in quarantine. This situation has now vastly changed, with many people experiencing long-term lockdown and quarantine, and the effects of this on pets are yet to be assessed.

The most common difficulty, experienced by 20% of the respondents, was the access to veterinary care. Similarly, Bowen et al. [1] reported that, during the pandemic in Spain, the most common concern for cat owners was the access to veterinary care and medication (39.6%). Research is still ongoing on the SARS-CoV-2 in cats [3, 4, 8]. So far, they were found to shed the virus for up to five days after infection and infect other cats by direct contact [3] and show measurable SARS-CoV-2 neutralizing antibody titres [8]. Information of the role of SARS-Cov-2 infected asymptomatic cats and other pets in dissemination of the virus will need to be re-evaluated over time, along with the potential consequences to the animals. Some papers recommend that domestic cats should be kept indoors to prevent them from interacting with other animals or people [17]. Clearly, multidisciplinary *One Health* and *One Welfare* approaches will be critical to developing appropriate strategies to effectively prevent and control the spread of COVID-19 in humans and animals [2, 18, 19].

In the first months of COVID-19 pandemic, animal shelters globally experienced an increase in adoption and foster rates [20], however, in the survey only two respondents re-homed their cat (one permanently). It may be that people who disposed their cat were not inclined to fill out a survey. The question remains open as to the future tendency as the pandemic continues. Difficulties may be considered as disadvantages of keeping cats during pandemic, since they involve more time or more endeavours devoted to resolve the difficulties, or fear of being infected. The respondents might experience other difficulties, disadvantages or inconveniences not listed in our survey, as indicated by 6% of the respondents. Some behavioural changes might be perceived both as an advantage and disadvantage. For example, an increased contact seeking of pet cats may be considered as advantageous for people who have more free time being in lockdown and are looking for amusement e.g., by playing with their cats, but may be considered disadvantageous for people who are busy with working of studying remotely (online) since a cat may be disturbing them [21, 22].

Even facing the possible risk of COVID-19 transmission by cats, people participating in the survey were reluctant to limit physical contact with cats owned by them, and also indicated that the greatest advantage of having a cat was the possibility of the reduction of emotional tension during a pandemic through contact. The majority of the respondents experienced benefits of having a cat during the pandemic, which is similar to previous surveys on cats [1] and dogs [1, 11]. Stammbach and Turner [23] found that human social support and owner attachment to the cat were interrelated. Their results indicated that for some of the survey participants, their cats can substitute for other humans in their social network [23]. In most cases, cats appear to be an additional source of emotional support [24], especially for those people who are strongly attached to their animals. Therefore, both social support and attachment are at work in these relationships, the relative importance of each depending upon the individual person in question.

We found that cat owners more often reported reduced tension due to the presence of the cat than dog owners (which was 65% for dog owners in a similar survey [11]). This is opposite to other studies on dog and cat human-animal relationships [25–28]. This may be related to the more positive behavioural changes reported in cats as compared to dogs during the pandemic [1, 11]. Overall, cats seem to cope better with the confinement during the pandemic than dogs, as reported by their owners, [1] and this may in turn contribute to the owner finding more support in their pet.

Most of the respondents owned one adult cat, which was mainly kept for longer than a year before the pandemic. This means that in current study most cats were already well socialised with their owners. McCune [29] found evidence that early experience with humans during the sensitive phase for socialisation had long-lasting effects well into adulthood. When the cats arrived in the household did not significantly affect any of the variables of interest but did improve the model fits. This was mainly due to the households with cats that arrived less than a month before the pandemic indicating numerically more behavioural changes and difficulties, but with a large variation which caused it to be non-significant (lower boundary of odd ratio below 1). Breed had no influence in the current study. In a large-scale survey to detect breed differences, Salonen and colleagues [30] found that British shorthair cats had the highest probability for reduced contact with people, the lowest probability for aggression, and were least active, as compared to a range of other cat breeds. Breed is therefore still recommended to take into consideration.

In this study, we faced a low response rate despite posts on social media and through emailing a broad network of animal researchers. The survey was available online for two months during the peak of the first wave of the pandemic. This particular restrictive period had been chosen since it could not be predicted how long the pandemic would last, whether the following waves of the pandemic would have happened, and what further preventative measures would be taken that may affect respondents’ perspectives and thus the study outcomes. Similar surveys on pet dogs have received vastly higher responses, e.g., 6004 dog owners in a survey by Dogs Trust [31], 2254 pet owners in the US [22], and 688 responses to a survey among dog owners with the same level of advertisement [11]. The large organisations that conducted these surveys will have had a wider reach and email data base than our small research team, and this will have influenced how many people were reached. The better response rate for dog surveys may also reflect different attitudes of people to their pet cat as compared to their dog. Within the low response rate we faced an uneven geographical distribution of the surveys. This may reflect the varying attitudes to cats as pets in different cultures or geographical regions.

The low response rate, which can bias data by providing a sample which is not representative of the population, can partly be explained by an increasing tendency to nonresponse to any type of survey, which has been observed worldwide in the last decades [32]. Main reasons for nonresponse are, as assessed by Brick and Williams [33], a lack of interest, being too busy, and time constraints. However, our survey took no longer than ten minutes to complete, was easy, anonymous (thus avoiding bias due to the request for sensitive information), and of potential interest to a large population of cat owners. It is still unclear how nonresponse produces a bias and decreases the accuracy of survey estimates [32]. In the current study, it could be that the respondents were more devoted to cat care and concerned with cat welfare, and that the non-responders might have had different interests and opinions. Although theoretically survey nonresponse may produce a bias [32], Hendra and Hill [34] suggest a scant relationship between survey nonresponse bias and response rates. Tourangeau and Plewes [32] also found little correlation between nonresponse rate and measures of bias and found no proof that efforts to enhance response rates within the context of a survey will automatically reduce nonresponse bias on survey estimates. In their extensive analysis of the nonresponse problem, they admit that actual non-response bias is still elusive [32], and suggest that low response rate means only that the potential for nonresponse bias has increased, and not necessarily that nonresponse bias has become a greater problem. The growing problem of nonresponse bias in surveys is, however, believed to decrease confidence in survey inference which is in contrast to the recent rapid growth in the use of surveys [32]. Although the total number of responses was low, the results are in line with previous similar surveys on cats and pet dogs, and thereby provide additional support for the research on the effects of the COVID-19 pandemic on animals and their caretakers.

## Conclusions

Our survey has shown that the COVID-19 pandemic did not create many difficulties for cat owners in regard to cat care and did not impact cats’ behaviour, as reported by their owners. Mostly positive behavioural changes were reported. Furthermore, pet cats contributed substantially to a reduction of psychological tensions in their owners during the pandemic. These conclusions are based on the situation up to June 2020. The continued effects of the pandemic, combined with the rapidly increasing scientific information on virus transmission by cats, call for the results to be followed up to understand changes in human-animal interaction and the welfare of pet animals after a prolonged exposure to the pandemic.

## Supporting information

S1 File Survey. (PDF)

S2 File Statistical output. (PDF)
