# Supplementary material for: Cat owners’ perception on having a pet cat during the COVID-19 pandemic
Source: PLoS One. 2021 Oct 20;16(10):e0257671. doi: 10.1371/journal.pone.0257671 (PMC8528273; doi:10.1371/journal.pone.0257671)
Supplement: S1 File — (PDF) [file pone.0257671.s001.pdf]

Supplementary files

Cat owners’ perception on having a pet cat during the COVID-19 pandemic

Tadeusz Jezierski, Irene Camerlink, Rachel S. E. Peden, Jen-Yun Chou, Patryk Sztandarski, Joanna Marchewka

Supplementary file 1. Survey

1. **In which country were you living during the pandemic?** [text box or country choice]

2. **How many cats do you have?** Tick the number of cats per age category

	1	2	3	4	5	6
Kitten (< 6 months of age)						
Adult (6 months – 12 years of age)						
Old (> 12 years of age)						

3. **What is the breed? If you have more than one cat then list the breeds of all cats** [text box]
4. **When did the most recent cat arrive at your household, counting from the start of the pandemic?**
   - Less than a month before the pandemic
   - Less than 6 months before the pandemic
   - 6 – 12 months before the pandemic
   - More than a year before the pandemic
5. **During the pandemic, where did the cat(s) mostly stay?**
   - Outdoors
   - Indoor, but not on places usually occupied by humans (bed, armchair etc.)
   - Indoor, including on places usually occupied by humans
6. **How frequent was the physical contact with the cat(s) during the pandemic?**
   - Only minimal touching, stroking or petting
   - 1-2 times a day briefly touching, stroking or petting
   - Multiple times a day touching, stroking or petting
7. **Did the care of the cat(s) change during the pandemic?**
   - No, care remained as usual
   - Yes, friends, relatives or neighbours took care of one or more of the cats
   - Yes, the cat(s) went to a shelter / animal hotel
8. **If yes, what was the reason for changing care?**
   - Fear of being infected with COVID-19
   - Difficulties with life organization due to restrictions during the pandemic
   - Financial difficulties due to the pandemic
   - Other

9. **Did you take any special measures or actions with respect to your cat(s) in order to prevent spreading of COVID-19?** Multiple answers possible.
   - No
   - Decreasing physical contact with the cat(s) compared to before
   - Letting the cat(s) less frequently outdoors
   - No more outdoor access beyond the boundaries of the house / garden
   - Disinfection of paws/coat
   - Avoiding the cat(s) to contact other people
   - Avoiding of close contact with other animals
   - Leaving of your cat(s) temporarily under custody of other people
   - Giving up the cat(s) forever
   - Other

10. **Did you observe any changes in the <u>behaviour</u> of your cat(s) during the pandemic as compared to before the pandemic?** *Exclude normal behavioural changes related to the season.* Multiple answers possible.
    - No change
    - Calmer
    - Anxiety
    - Apathy
    - Avoiding a close contact to caretaker (e.g. hiding)
    - More frequent seeking close contacts (approaching, allowing petting etc.)
    - Development or increase of repetitive (stereotypic) behaviour
    - Increased aggression towards humans
    - Increased damaging behaviour (e.g. scratching door)
    - Incontinence / urinating or defecating indoors, outside the litter box
    - More frequent meowing/vocalizations
    - Less frequent meowing/ vocalizations
    - More frequent requests for going outdoors
    - Reluctance of being let outdoors
    - More playful
    - Less playful
    - Restlessness
    - Other behavioural changes

11. **Did you observe any changes in the <u>health</u> of your cat(s) during the pandemic as compared to before the pandemic?** Multiple answers possible.
    - No change
    - Constipation
    - Diarrhoea
    - Decreased mobility
    - Decreased appetite
    - Increased appetite
    - Skin problems
    - Other

12. **Does your cat suffer from any chronic disease?**
    - Yes, skin/parasitic diseases
    - Yes, internal diseases
    - Yes, other chronic disease
    - No

13. **Which difficulties did you experience of keeping a cat during the pandemic?**
    - No difficulties
    - Fear of being infected with COVID-19 because of reasons related to the cat
    - Food supply
    - More difficult access to veterinary care

- Unfavourable changes in cat's behaviour
- Other difficulties

14. **Did you see any advantages of the presence of a cat during the pandemic?**
    - Yes, reduction of own psychological tensions because of contact with cat(s)
    - Yes, desirable changes in cat's behaviour
    - Other advantages
    - No advantages
15. **What best describes where you lived most of the time during the pandemic?** Tick all that apply.
    - Densely populated city / capital city
    - Regular city
    - Suburb/small town
    - Country side
    - Flat without yard/garden
    - House/apartment with small yard/garden
    - House/farm with big yard/garden
16. **How many adult persons were staying at the house during the pandemic?** [text box]
17. **Did you have to take care of children during the pandemic**?
    - Yes, one or more infants of < 1 years old
    - Yes, one or more children of 1 – 5 years old
    - Yes, one or more children of 5 – 15 years old
    - No
18. **For how long have you been restricted in going outside (lock-down)?** Tick what best applies.
    - Not at all
    - 2 weeks
    - 1 month
    - 2 months
    - 3 months
    - More than 3 months
19. **Have you or anyone in your household been under quarantine?**
    - Yes, for 2 weeks
    - Yes, for more than 2 weeks
    - No
    - Prefer not to disclose
20. **What is your gender?**
    - Man
    - Woman
    - Prefer not to disclose
21. **What is your year of birth?** [text box]
