# Supplementary material for: Cat owners’ perception on having a pet cat during the COVID-19 pandemic
Source: PLoS One. 2021 Oct 20;16(10):e0257671. doi: 10.1371/journal.pone.0257671 (PMC8528273; doi:10.1371/journal.pone.0257671)
Supplement: S2 File — (PDF) [file pone.0257671.s002.pdf]

Supplementary file S2. Statistical output

Behavioural changes in the cat (yes/no)

Variable	Full model	Reduced model*	Best fitting model
Children in household (y/n)	0.9026		
Arrival of cat in household	0.3363		0.3472
Cat's age	0.8956		
Measures taken to prevent virus (y/n)	0.0328	0.0872	0.0468
Restriction level (lockd./quadrant./contr.)	0.0928	0.0658	0.1020
One cat or more (y/n)	0.2991		
**Test statistics**			
AIC (smaller is better)	411.85	410.22	406.74
AICC (smaller is better)	412.87	410.40	407.20
BIC (smaller is better)	426.47	416.31	416.49

*model only including p-values <0.10

Difficulties of caring for a cat during the pandemic (yes/no)

Variable	Full model	Reduced model*	Best fitting model
Children in household (y/n)	0.2878		0.2138
Arrival of cat in household	0.0631	0.0713	0.0603
Cat's age	0.2692		
Measures taken to prevent virus (y/n)	<.0001	<.0001	<.0001
Restriction level (lockd./quadrant./contr.)	0.2399		
One cat or more (y/n)	0.3836		
**Test statistics**			
AIC (smaller is better)	358.63	355.75	354.44
AICC (smaller is better)	359.65	356.02	354.80
BIC (smaller is better)	373.25	363.07	362.97

*model only including p-values <0.10

Advantages of having a cat (yes/no)

Variable	Full model	Reduced model	Best fitting model
Children in household (y/n)	0.0758	0.0660	0.0660
Arrival of cat in household	0.8237		
Cat's age	0.6096		
Measures taken to prevent virus (y/n)	0.8133		
Restriction level (lockd./quadrant./contr.)	0.2337		
One cat or more (y/n)	0.2311		
**Test statistics**			
AIC (smaller is better)	280.62	273.87	273.87
AICC (smaller is better)	281.64	273.95	273.95
BIC (smaller is better)	295.25		277.53
